# Clinical factors associated with stable treatment of chronic constipation in Japanese patients

**DOI:** 10.1186/s12876-024-03140-y

**Published:** 2024-01-29

**Authors:** Kazuki Ishibashi, Yuji Urabe, Nhu Thi Hanh Vu, Shunsuke Miyauchi, Takeo Nakamura, Hirona Konishi, Junichi Mizuno, Motomitsu Fukuhara, Hidehiko Takigawa, Ryo Yuge, Duc Trong Quach, Shiro Oka, Toru Hiyama

**Affiliations:** 1 Department of Gastroenterology, Graduate School of Biomedical and Health Sciences, Hiroshima, Japan; 2 Department of Gastrointestinal Endoscopy and Medicine, Hiroshima University Hospital, Hiroshima, Japan; 3 Department of Internal Medicine, University of Medicine and Pharmacy at Ho Chi Minh City, Ho Chi Minh, Vietnam; 4 Health Service Center, Hiroshima University, 1-7-1 Kagamiyama, 739-8514 Higashihiroshima, Japan

**Keywords:** Chronic constipation, Stable treatment, Unstable treatment, Clinical factor, Acid-suppressive drugs

## Abstract

**Background/Aims:**

Chronic constipation (CC) is one of the most common gastrointestinal disorders in the general population. Although there are many treatment options, achieving a stable treatment for CC remains one of the challenges in clinical practice. This study aimed to evaluate the clinical factors associated with stable treatment for CC in Japanese patients.

**Methods:**

A retrospective, cross-sectional, and multicenter study was carried out. Patients were eligible for inclusion if they fulfilled the Rome IV criteria for diagnosing CC and had been treated for at least one and a half years. Patients with up to two prescription modifications for CC in one year were defined as the stable treatment group, whereas those with three or more prescription changes were defined as the unstable treatment group. Univariate and multivariate analyses were carried out to identify factors associated with CC.

**Results:**

A total of 114 patients have been recruited. There were 82 patients (77.0%) in the stable treatment group and 32 patients (23.0%) in the unstable treatment group. Based on multivariate likelihood analysis, only using acid-suppressive drugs contributed to stability treatment in CC patients (odds ratio: 2.81, 95% confidence interval: 1.12–7.08, *p* = 0.03).

**Conclusion:**

Administration of acid-suppressive drugs was the only factor related to the stability of CC treatment. Further studies are needed to validate the results as well as clarify the causes.

## Background

Chronic constipation (CC) is one of the most common gastrointestinal disorders in the general population [1]. In Japan, the prevalence of CC is estimated to be 6.1–28%, depending on the diagnostic scale utilized [2, 3]. CC is characterized by unsatisfactory defecation due to infrequent bowel movements and/or difficulty defecating [4]. Defecation is a physiological phenomenon, and its disturbance can be a severe problem in daily life, causing not only physical symptoms but also psychological symptoms.

Several studies demonstrated that CC has significant deleterious effects on daily life, impairs health-related quality of life, and may impose a considerable social and economic burden [5–7]. Therefore, appropriate treatment and maintaining the patient’s stable condition are critical in treating CC. Previously, osmotic laxatives and stimulant laxatives were commonly used in Japan, but recently epithelial function-altering drugs, including lubiprostone, linaclotide, and elobixibat have also become increasingly popular [6–8]. Thus, therapy options for CC have been expanded, and treatment goals may be attained more readily.

Although there are many treatment options, achieving a stable treatment for CC remains one of the challenges in clinical practice. To the best of our knowledge, there is a lack of published data on the variables that affect CC treatment stability. Hence, this study aimed to evaluate the clinical factors associated with stable CC treatment in Japanese patients.

## Methods

### Study participants

This retrospective, cross-sectional, and multicenter study was carried out in Japan from January to March 2022. Patients with CC who visited Hiroshima University Hospital (Hiroshima, Japan), Kawamura Internal Medicine Clinic (Hiroshima, Japan), and Matsuonaika Hospital (Mihara, Japan) were eligible for inclusion if they were at least 18 years of age, fulfilled the Rome IV criteria [4] for the diagnosis of CC, and had been treated CC for at least one and a half year. Patients were excluded if they had one or more of the following conditions: inflammatory bowel diseases such as ulcerative colitis or Crohn’s disease, gastrointestinal malignancies, pregnancy, secondary constipation, drug-induced constipation, not willing to participate in the study. All physicians participating in the study were gastroenterologists. We made a treatment plan based on various factors, including the patient’s reported Bristol Stool Form Scale type, bowel frequency, and satisfaction with the treatment.

### Diagnostic criteria of CC

According to the Rome IV criteria [4], the diagnosis of CC was made when at least two of the following conditions were present: (1) straining during more than one-fourth (25%) of the defecations; (2) lumpy or hard stools (Bristol stool form [9] scale 1 or 2) in more than one-fourth (25%) of the defecations; (3) a sensation of incomplete evacuation in more than one-fourth (25%) of the defecations; (4) a sensation of anorectal obstruction/blockage in more than one-fourth (25%) of the defecations; (5) performance of manual maneuvers (digital evacuation or pelvic floor assistance) to facilitate more than one-fourth (25%) of the defecations; and (6) fewer than three spontaneous bowel movements per week. The above criteria must have been fulfilled during the last three months, with the onset of symptoms occurring at least six months prior to diagnosis.

### Definition of stable and unstable treatment groups

Patients were divided into two groups: stable and unstable treatment groups. Patients were classified as unstable if their initial laxative therapy progressed through the switch, augmentation, or dose adjustment [10]. Patients with up to two prescription modifications for CC in one year were defined as the stable treatment group, whereas those with three or more prescription changes were defined as the unstable treatment group. All patients were observed for six months prior to categorizing them as having stable or unstable constipation because some patients had to alter their medication regimens within the initial six months following treatment initiation. This helps limit confounding factors and provides a more rigorous definition of constipation stability.

### Data collection

The following items were extracted from the electronic medical records: age, sex, body mass index (BMI), history of abdominal surgery, hemoglobin, liver function, kidney function, past history (diabetes, thyroid dysfunction, mental disorders, dementia, Parkinson’s disease), use of acid-suppressive drugs, use of prokinetic drugs, and use of 3 or more types of drugs for CC. The most recent blood test results were utilized in the study.

Obesity was determined as a BMI ≥ 30 kg/m^2^, while underweight was defined as a BMI ≤ 18.5 kg/m^2^ [11]. Anemia was identified as hemoglobin below 13 g/dL for males and below 12 g/dL for females. Abnormal liver function was described if AST was greater than 36 U/L or ALT was greater than 41 U/L. Abnormal kidney function was reported if creatinine levels were ≥ 1.1 mg/dL in males and ≥ 0.9 mg/dL in females. Acid-suppressive drugs included histamine-2 receptor antagonists (H_2_RA) (famotidine), proton pump inhibitors (PPIs) (esomeprazole, lansoprazole, omeprazole, and rabeprazole), and potassium-competitive acid blockers (vonoprazan). Prokinetic drugs included acotiamide, mosapride, pantethine, and rikkunshito. Short-term (less than six months) administration of acid-suppressive agents was ruled out. Laxatives included magnesium oxide, polyethylene glycol/electrolyte solution, sennoside, senna, bisacodyl, sodium picosulfate, sodium bicarbonate, lubiprostone, linaclotide, and elobixibat.

### Statistical analysis

All statistical analyses were performed using SPSS software version 20.0 (SPSS Inc., Chicago, IL, U.S.A.). Continuous variables were summarized as means (± SD), whereas categorical variables were summarized as proportions or percentages. The student’s t-test was used to evaluate the continuous variables. Categorical variables were analyzed using χ2 and Fisher’s exact tests as appropriate. Univariate and multivariate analyses were carried out to identify factors associated with CC. We used logistic regression models for multivariable analyses to identify clinical factors and to calculate odds ratios (ORs) and 95% confidence intervals. *p* values < 0.05 were considered statistically significant.

## Results

### Clinical features of study participants

There were 114 patients who met the criteria for inclusion. The clinical features of the patients are presented in Table 1. The mean age of patients was 76.7 ± 13, with a range of 33–101 years. The ratio of male-to-female was 1:1.67. Nineteen patients (16.7%) needed three or more types of laxatives for the treatment of CC. The rate of patients with mental disorders and dementia was 13.2% and 5.3%, respectively.

**Table 1:** Clinical characteristics of studying patients and the comparison between the stable treatment and unstable treatment group

	Stable treatment	Unstable treatment	Total	p-value
	(*n* = 82)	(*n* = 32)	(*n* = 114)	
Age (mean ± SD)	76.9 ± 13.3	78.2 ± 10.8	77.3 ± 12.7	0.65
Over 70 years old, n (%)	62 (75.6)	28 (87.5)	90 (78.9)	0.16
Male (n, %)	32 (39)	14 (43.8)	46 (40.4)	0.64
Obesity (n, %)	5 (6.1)	1 (3.1)	6 (5.3)	0.52
Underweight (n, %)	10 (12.2)	1 (3.1)	11 (9.6)	0.14
History of abdominal surgery (n, %)	38 (46.3)	16 (50)	54 (47.4)	0.73
Anemia (n, %)	43 (52.4)	12 (37.5)	55 (48.2)	0.15
Abnormal liver function (n, %)	10 (12.2)	6 (18.8)	16 (14)	0.37
Abnormal kidney function (n, %)	33 (40.2)	7 (21.9)	40 (35.1)	0.07
Diabetes mellitus (n, %)	32 (39)	8 (25)	40 (35.1)	0.16
Hypothyroidism (n, %)	7 (8.5)	3 (9.4)	10 (8.8)	0.89
Mental disorders (n, %)	13 (15.9)	2 (6.2)	15 (13.2)	0.17
Dementia (n, %)	4 (4.9)	2 (6.2)	6 (5.3)	0.77
Parkinson’s disease (n, %)	1 (1.2)	0	1 (0.9)	0.53
Use of acid-suppressive drugs (n, %)	56 (68.3)	13 (40.6)	69 (60.5)	0.007
Use of prokinetic drugs (n, %)	11 (13.4)	2 (6.2)	13 (11.4)	0.28
Use of 3 or more types of laxatives (n, %)	12 (14.6)	7 (21.9)	19 (16.7)	0.35

### Association between clinical characteristics and treatment status for chronic constipation

There were 82 patients (77.0%) in the stable treatment group and 32 (23.0%) in the unstable treatment group. The only significant difference was the use of acid-suppressive medications between the two groups (68.3% for the stable treatment group vs. 40.6% for the unstable treatment group, *p* < 0.05). Of the 82 patients in the stable group, 56 patients (68.3%) were using acid-suppressive drugs, including vonoprazan (6 patients), PPIs (45 patients), and H_2_RA (5 patients). On the other hand, 13 (40.6%) of the 32 patients in the unstable group were using acid-suppressive drugs, including vonoprazan (1 patient), PPIs (10 patients), and H_2_RA (10 patients). No other differences were found in the univariate analysis of all other clinical factors (Table 1).

Based on multivariate likelihood analysis, only the use of acid-suppressive drugs contributed to stability treatment in patients with CC (odds ratio (OR): 2.81, 95% confidence interval (CI): 1.12–7.08, *p* = 0.03) (Table 2).

**Table 2:** Summary of multivariable logistic regression analysis

Clinical factors	Multivariable		
	OR	95% CI	p-value
Over 70 years old	0.34	0.1–1.22	0.1
Underweight	2.74	0.3-24.84	0.37
Anemia	1.36	0.53–24.84	0.53
Abnormal kidney function	2.45	0.87–6.88	0.09
Diabetes mellitus	2.19	0.81–5.95	0.13
Mental disorders	1.92	0.37–9.91	0.43
Use of acid-suppressive drugs	2.81	1.12–7.08	0.03

OR: odds ratio; CI: confidence interval

## Discussion

This study suggests that using acid-suppressive drugs may be a significant factor associated with the stability of CC treatment. No prior studies have evaluated the correlation between utilizing acid-suppressive medications and stable treatment in patients with CC. There are two possible reasons. The first reason is that the overlap of functional gastrointestinal disorders is common in patients with CC, particularly functional dyspepsia (FD) and gastroesophageal reflux disease (GERD) [12, 13]. A prospective nationwide multi-center study of 759 CC patients in Korea showed that 10.5% of CC patients had FD overlap, 17.9% had overlap GERD, and 6.7% had both GERD and FD overlap [13]. Many studies have demonstrated the efficacy of acid-suppressive drugs for the treatment of FD and GERD, regardless of the dose and duration of treatment compared to placebo [14–16]. As a result, improving FD and GERD symptoms with acid-suppressive drugs may also reduce the symptoms of CC and contribute to the stability of treatment in CC patients. Moreover, functional gastrointestinal disorders, including FD and CC are associated with and accompanied by mental disorders such as depression, and emotional stress [17, 18]. In our study, the stable treatment group had more patients with mental disorders than the unstable treatment group. Therefore, alleviating FD symptoms with acid-suppressive drugs may reduce stress levels and stabilize CC treatment. This can be explained by several reasons. The gut-brain axis connects the gastrointestinal tract and the brain [19]. When the gastric produces excessive acid, it can lead to physical discomfort and contribute to stress and anxiety. Acid-suppressing drugs may improve these physical symptoms by reducing gastric acid production, which assists in reducing stress levels. Additionally, it has been demonstrated that there may be a direct relationship between gastric acid and stress hormones. Excessive gastric acid can stimulate the production of stress hormones, such as cortisol [20]. As a result, reducing gastric acid may have the opposite effect and decrease stress levels.

Another reason is that acid-suppressive drugs may affect the intestinal bacteria that stabilize CC therapy. The intestinal microbiota is a collection of microorganisms that reside in the gastrointestinal tract and have recently been shown to conduct many critical health-promoting functions [21, 22]. It is known that the number of gastrointestinal bacteria in CC patients is approximately 10 times higher than the number of cells in the human body, with up to 1000 different bacterial species [23]. Alterations of intestinal microbiota in patients with CC can be characterized by a relative decrease in obligate bacteria (e.g. *Lactobacillus, Bifidobacterium and Bacteroides spp*) and a parallel increase in potentially pathogenic microorganisms (e.g. *Pseudomonas aeruginosa and Campylobacter jejuni*) [24, 25]. These alterations may affect intestinal motility and secretory functions by changing the amount of available physiologically active substances and the metabolic environment of the gut, and may be one of the causes of constipation. It has been reported that using proton-pump inhibitors, one of the acid-suppressive drugs, increases the number of oral commensal bacteria in the intestinal microbiota and alters the intestinal microbiota [26–28]. Therefore, it is possible that alterations in the intestinal microflora induced by the use of acid-suppressive drugs contributed to the stabilization of constipation treatment.

In our study, 11.4% of patients were prescribed prokinetic medications. Prokinetic agents enhance gastrointestinal motility and are considered clinically relevant in the treatment of functional gastrointestinal disorders characterized by impaired motility, including FD and CC [29]. The use of prokinetics in the stable treatment was higher than that in the unstable treatment groups; however, there was no statistically significant difference.

Previous studies have shown that constipation has been associated with increasing age and comorbidities such as diabetes and mental health [1, 30–33]. Contrary to our expectations, we did not identify a statistically significant difference in CC treatment stability with these comorbidities. Another prospective cohort study over 20 years found that patients with persistent and non-persistent CC have similar clinical characteristics, although those with persistent CC use more laxatives or fiber [34]. In our study, more patients in the unstable treatment group received more than three types of laxatives. However, there was no statistically significant difference. Further research would be necessary to examine these correlations.

According to our results, many patients were in the geriatric group and had comorbidities such as dementia, hypothyroidism, and psychiatric disorders. These comorbidities have been reported to induce constipation; however, multivariate analysis in this study did not identify any association between these factors and stabilized CC treatment.

This study has several limitations. First, our study had a limited number of cases and participating institutions, as well as the different distribution of numbers between the two groups. Due to the small size, we can not address the type of acid-suprressive in our study. The main reason for the small study size was that the number of patients continuously observed and treated for one and a half years in each medical facility was not so many. Second, medical history and comorbidities were retrospectively analyzed based on medical records, which may have been insufficient. For example, we do not have detailed information on other potentially confounding items such as antibiotic and supplement use, alcohol, and smoking history. Third, medication modifications were left to the judgment of each treating physician, with no standardized criteria. The decision to switch prescriptions was mainly based on the patient’s subjectively reported symptoms and level of satisfaction, both of which may have been subjective. Fourth, the period time to assess whether the treatment is stable or not in our study is relatively short. More clinical factors contributing to treatment stability may become apparent with additional time. Fifth, we do not have enough information to diagnose the patients with FD, GERD, and the overlapping between CC and FD/GERD, as well as irritable bowel syndrome with constipation. This would be a crucial research question for future studies of our group.

## Conclusion

In conclusion, the administration of acid-suppressive drugs was the only factor related to the stability of CC treatment. Further studies are required to validate the findings as well as clarify the causes.

## Data Availability

All data generated during this study are included in this article. Further inquiries can be directed to the corresponding author.

## Abbreviations

CC: Chronic constipation
OR: odds ratio
CI: confidence interval
